# *In vivo* reprogrammed pluripotent stem cells from teratomas share analogous properties with their *in vitro* counterparts

**DOI:** 10.1038/srep13559

**Published:** 2015-08-28

**Authors:** Hyun Woo Choi, Jong Soo Kim, Yean Ju Hong, Hyuk Song, Han Geuk Seo, Jeong Tae Do

**Affiliations:** 1 Department of Animal Biotechnology, College of Animal Bioscience and Technology, Konkuk University, Seoul, 143-701, Republic of Korea

## Abstract

Recently, induced pluripotent stem cells (iPSCs) have been generated *in vivo* from reprogrammable mice. These *in vivo* iPSCs display features of totipotency, i.e., they differentiate into the trophoblast lineage, as well as all 3 germ layers. Here, we developed a new reprogrammable mouse model carrying an *Oct4*-GFP reporter gene to facilitate the detection of reprogrammed pluripotent stem cells. Without doxycycline administration, some of the reprogrammable mice developed aggressively growing teratomas that contained *Oct4*-GFP^+^ cells. These teratoma-derived *in vivo* PSCs were morphologically indistinguishable from ESCs, expressed pluripotency markers, and could differentiate into tissues of all 3 germ layers. However, these *in vivo* reprogrammed PSCs were more similar to *in vitro* iPSCs than ESCs and did not contribute to the trophectoderm of the blastocysts after aggregation with 8-cell embryos. Therefore, the ability to differentiate into the trophoblast lineage might not be a unique characteristic of *in vivo* iPSCs.

Enforcing the expression of a specific combination of reprogramming factors such as *Oct4*, *Sox2*, *Klf4*, and *c*-*Myc* (*OSKM*) in somatic cells can result in the generation of induced pluripotent stem cells (iPSCs). These iPSCs express pluripotent stem cell markers and can differentiate into cells of all 3 germ layers and germ cells *in vitro* and *in vivo*. iPSCs have been suggested as a substitute for embryonic stem cells (ESCs) because of their similar differentiation potentials; moreover, iPSCs can be generated without sacrificing embryos. iPSCs can also be generated from diverse cell types, including extraembryonic^1^ and uniparental cell sources^2^. Although iPSCs are functionally very similar to ESCs, they are not identical and can be distinguished on the basis of global gene expression patterns^3^. Therefore, many researchers are constantly striving to generate iPSCs that more closely resemble ESCs. Non-viral reprogramming systems have been applied for iPSC generation, such as episomal introduction of DNA or RNA, protein delivery, and the sole use of small molecules^4^,^5^,^6^,^7^,^8^,^9^. High-quality naïve pluripotent iPSCs have been generated by cultivation in media supplemented with vitamin C or the 2-inhibitor (2i) culture condition (mitogen-activated protein kinase and glycogen synthase kinase 3 inhibitors)^10^,^11^,^12^.

Considerable progress has been made in direct reprogramming under *in vitro* culture conditions^13^. However, recent reports have suggested that the *in vivo* environment might serve as the niche for direct reprogramming. A series of studies have shown that the overexpression of fate-determining genes *in vivo* could reprogram resident cells or injected cells into other cell types, including neurons and neuroblasts^14^,^15^,^16^. Injection of plasmids encoding *OSKM* into the tail vein induced the upregulation of pluripotency-related genes in hepatocytes without subsequent teratoma formation^17^. iPSCs have also been generated *in vivo* from circulating blood cells of reprogrammable mice^18^,^19^. These *in vivo* iPSCs resemble ESCs more closely than iPSCs generated *in vitro*. Moreover, *in vivo* iPSCs differentiate into the trophectoderm lineage and all 3 germ layers, thereby demonstrating totipotency. However, differentiation into the trophoblast lineage is not characteristic of naïve pluripotent ESCs but is a primed pluripotency feature, as human ESCs preferentially differentiate into the trophoblast lineage^20^. These facts prompted us to address more characteristics from new *in vivo* iPSC lines. Here, we have developed a new method to generate *in vivo* iPSCs, which can be easily selected from reprogrammable mice. To facilitate the detection of reprogrammed cells, we generated a triple transgenic mouse carrying a transcriptional activator (rtTA; within the ubiquitously-expressed Rosa26 locus), a doxycycline-inducible polycistronic cassette encoding the 4 reprogramming factors (OSKM) within the *Col1a1* locus, and *Oct4*-GFP. We obtained *Oct4*-GFP^+^ cells from teratomas of the reprogrammable mice. The reprogrammed PSCs were established from these teratoma-derived *Oct4*-GFP^+^ cells, which were morphologically indistinguishable from ESCs. However, these *in vivo* reprogrammed PSCs (rPSCs) were more similar to *in vitro* iPSCs than ESCs and did not contribute to the trophectoderm of the blastocysts after aggregation with 8-cell embryos. Therefore, differentiation ability into the trophoblast lineage might not be a unique characteristic of iPSCs derived from the *in vivo* milieu.

## Results

### Generation of reprogrammable mice with Oct4-GFP

The reprogrammable mouse is a useful tool to study the mechanisms underlying cellular reprogramming triggered by doxycycline administration^19^. We generated reprogrammable mice, which were the F1 generation of reprogrammable mice crossed with OG2 mice, which carry the *Oct4*-GFP (ΔPE) transgene. However, mouse embryonic fibroblasts (MEFs) from these heterozygous reprogrammable mice (OG2^+/−^/RTC4^+/−^) were rarely reprogrammed to *Oct4*-GFP^+^ cells after doxycycline treatment (data not shown). Reprogramming efficiency has been reported to be higher when the reprogrammable MEFs are homozygous for *OKSM* and *Rosa26-M2rtTA* transgenes (Ho/Ho) than when MEFs are heterozygous for each transgene (Het/Het)^21^. Thus, we generated a new set of reprogrammable mice homozygous for the transcriptional activator (M2rtTA; within the ubiquitously expressed Rosa26 locus) and doxycycline-inducible polycistronic cassette encoding the 4 reprogramming factors *OSKM* within the Col1a1 locus, and heterozygous for *Oct4*-GFP transgene (ΔPE)^19^. We named the reprogrammable mice rOG2 (for reprogrammable OG2) mice (Supplemental material Fig. S1).

First, we tested whether the rOG2 mice were successfully reprogrammable upon doxycycline induction. Neural stem cells (NSCs) and MEFs were obtained from rOG2 mice and cultured in doxycycline-containing ESC medium. At 10–15 days after culture with doxycycline, *Oct4*-GFP^+^ cells were observed. After further passage, the *Oct4*-GFP^+^ cells formed dome-like colonies, like ESCs, and were called rOG2-MEF-iPSCs (Supplemental material Fig. S3A and B). This result indicated that the reprogramming cassette and the *Oct4*-GFP transgene combination in rOG2 mice functioned properly in an *in vitro* system.

### Spontaneous generation of teratomas in rOG2 mice

**Figure 1:**
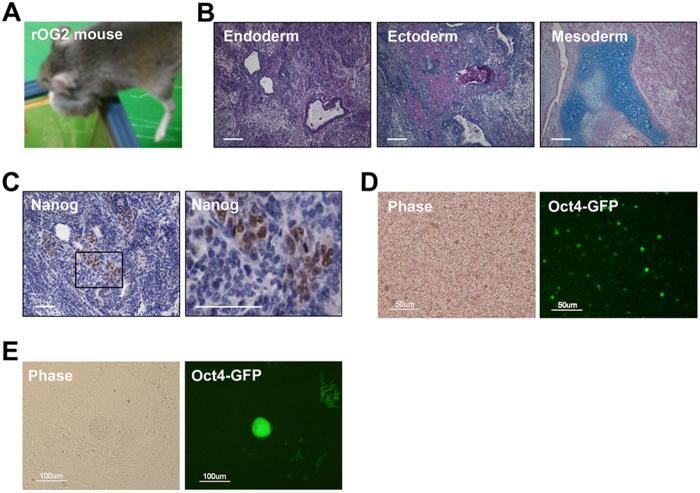
Generation of rOG2 mice and spontaneously forming teratomas. (**A**) Spontaneously forming teratomas in rOG2 mice at the age of 4 weeks without doxycycline treatment. (**B**) Teratomas of rOG2 mice contained all 3 germ layer-like structures. Scale bar = 100 μm. (**C**) The undifferentiated cells in teratomas expressed *Nanog*, as shown by immunohistochemistry. Scale bar = 100 μm. (**D,E**) *Oct4*-GFP–positive cells in the teratomas were observed (Scale bar = 50 μm), which were sorted and cultured in conventional ESC culture condition without doxycycline. Scale bar = 100 μm.

As previously reported, some reprogrammable mice spontaneously developed aggressively growing tumors that histologically presented as largely undifferentiated teratomas^19^,^22^. The rOG2 mice also formed teratomas spontaneously at the age of 4 weeks without doxycycline administration (Fig. 1A). Interestingly, these teratomas contained not only differentiated cell populations of all 3 germ layers (Fig. 1B) but also undifferentiated cell populations expressing *Nanog* (Fig. 1C), indicating that certain somatic cells in rOG2 mice were spontaneously reprogrammed into the pluripotent state *in vivo*. We dissociated the teratomas into single cells and examined them by fluorescence microscopy. Many *Oct4*-GFP^+^ cells were detected in the cells from teratomas (Fig. 1D and Supplemental material Fig. S2). The GFP^+^ cells could be pluripotent cells that were generated *in vivo*. The GFP^+^ cells were sorted by FACS and cultured on a feeder-layered dish in ESC culture medium. To our surprise, the GFP^+^ cells formed ESC-like colonies in ESC culture conditions (Fig. 1E). These ESC-like cells might be iPSCs induced by the *in vivo* environment (rOG2-T-rPSCs).

### Characteristics of the *in vivo* iPSCs

**Figure 2:**
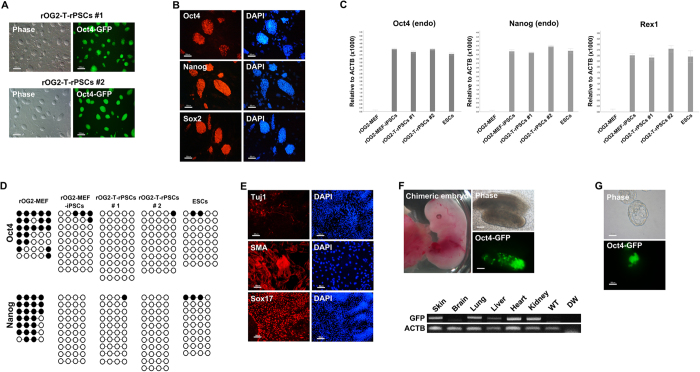
Pluripotency of *in vivo* rPSCs. (**A**) The established *in vivo* rPSCs expressed *Oct4*-GFP and were morphologically indistinguishable from mESCs. Scale bar = 100 μm. (**B**) Immunocytochemistry analysis (scale bar = 50 μm) and (**C**) qRT-PCR showed that *in vivo* rPSCs expressed pluripotency markers *Oct4* (endo), *Nanog* (endo), *Sox2*, and *Rex1*, similar to *in vitro* iPSCs and ESCs. (**D**) DNA methylation status of *Oct4* and *Nanog* promoter region in rOG2-T-rPSCs #1, #2. (**E,F**) The rOG2-T-rPSCs could differentiate into all 3 germ layers *in vitro* and *in vivo* Scale bar = 100 μm. (**G**) Aggregation potential of *in viv*o rPSCs into the inner cell mass of normal embryos. The *in vivo* rPSCs did not contribute to the trophectoderm lineage. Scale bar = 50 μm.

By clonal expansion, we established 2 *in vivo* reprogrammed PSC (rPSC) lines, designated as rOG2-T-rPSCs #1 and #2, from the FACS-sorted GFP^+^ cells (Fig. 2A). Immunocytochemistry analysis showed that these rOG2-T-rPSCs were positively stained for core pluripotency markers, *Oct4*, *Sox2*, and *Nanog* (Fig. 2B). qRT-PCR analysis also showed that *in vivo* rPSCs expressed endogenous pluripotency markers *Oct4* (endo), *Nanog* (endo), and *Rex1* at levels similar to those of *in vitro* iPSC and ESCs (less than twofold; Fig. 2B,C). Next, we investigated the DNA methylation status in *Oct4* and *Nanog* promoter regions of rOG2-T-rPSCs #1 and #2. *Oct4* and *Nanog* promoter regions were completely demethylated in rOG2-T-rPSCs #1 and #2, as shown in rOG2-MEF-iPSCs and ESCs (Fig. 2D). The rOG2-T-rPSCs could differentiate into all 3 germ layers *in vitro* via embryoid body formation (Fig. 2E) and could form germline chimeras (Fig. 2F). Thus, PSCs reprogrammed *in vivo* from reprogrammable mice possess pluripotent characteristics, including the overexpression of pluripotency marker genes, DNA demethylation in *Oct4* and *Nanog* promoter regions, and *in vitro* and *in vivo* differentiation potential. Next, to test the developmental potential to trophoblast lineage, we aggregated the *in vivo* iPSCs with 8-cell embryos and cultured them until the blastocyst stage. However, we could not observe the contribution of *in vivo* iPSCs to the trophectoderm of blastocysts (0/200) (Fig. 2G and Supplemental material Fig. S5).

### Gene expression pattern of *in vivo* iPSCs

**Figure 3:**
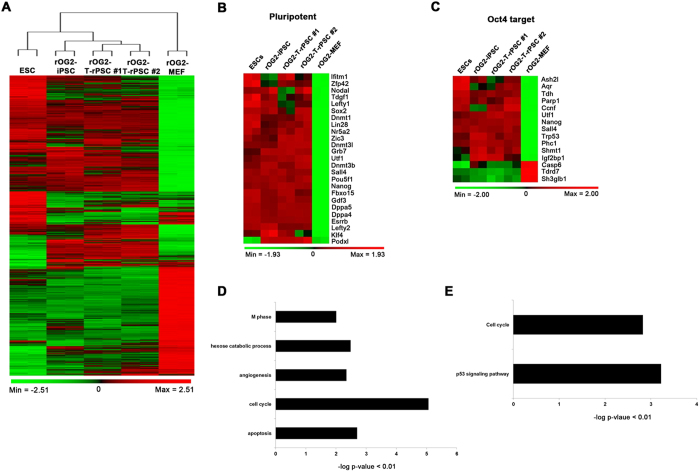
Gene expression pattern *in vivo* iPSCs. (**A**) Heatmap data showed that the gene expression pattern of *in vivo* rPSCs was similar to that of control mESCs. (**B,C**) The heatmap of pluripotency and *Oct4* target-related gene expression in ESCs, rOG2-MEF-iPSCs, rOG2-T-rPSCs #1 rOG2-T-rPSCs #2, and rOG2-MEF. (**D**) GO term and (**E**) KEGG pathway annotation using DAVID.

To compare the molecular signatures of the rOG2-T-rPSCs, we performed gene expression profiling by microarray analysis (Illumina’s MouseRef-8 v2 Expression BeadChip). Pearson correlation analysis was used to cluster the cells according to the gene expression profiles. Heatmap and hierarchical clustering analyses showed that the global gene expression patterns of rOG2-T-iPSCs #1 and #2 cells were similar to those of ESCs and rOG2-MEF-iPSCs (Fig. 3A). However, *in vivo* rPSCs were more similar to *in vitro* iPSCs than ESCs. Scatter plot analysis showed that the *r*^2^ values (square of linear correlation coefficient) between ESCs and rOG2-T-riPSCs #1 and #2 cells were 0.96–0.97 (Supplemental material Fig. S4). The expression of pluripotency markers and *Oct4* target genes in rOG2-T-rPSCs #1 and #2 was also closer to the rOG2-MEF-iPSCs than ESCs (Fig. 3B,C).

To further characterize the rOG2-T-iPSCs, we categorized the functions of the differentially expressed genes between rOG2-T-rPSCs and mESCs by Gene Ontology (GO) analysis. We isolated differentially expressed genes (more than a twofold change) and found that 415 probes were upregulated and 337 probes were downregulated in rOG2-T-rPSCs cells versus mESCs. We performed the GO term and KEGG pathway annotation using DAVID (http://david.abcc.ncif.gov/). According to GO analysis, upregulated genes in rOG2-T-rPSCs were enriched for the terms “apoptosis,” “cell cycle,” “angiogenesis,” “hexose catabolic process,” and “M phase” (p value < 0.01) (Fig. 3D, Supplemental material Table. 1), whereas downregulated genes in rOG2-T-rPSCs were enriched for “cell redox homeostasis” (p value < 0.001) (Supplemental material Table 2). In addition, pathway annotation of up- and downregulated genes was performed based on scoring and visualization of the pathways obtained from the KEGG database (http://www.genome.jp/kegg/). The upregulated genes in rOG2-T-rPSCs included “p53 signaling pathway” and “cell cycle” (p value < 0.01) (Fig. 3D,E, Supplemental material Table 3), whereas the downregulated genes included “propanoate metabolism,” “pyruvate metabolism,” “glycolysis/gluconeogenesis,” and “valine, leucine, and isoleucine degradation” (p value < 0.01) (Supplemental material Table 4).

## Discussion

We established *in vivo* reprogrammed PSC lines from teratomas formed in reprogrammable mice containing the *Oct4*-GFP marker. These *in vivo* rPSCs expressed pluripotency markers, displayed an epigenetic status similar to that of ESCs, and could differentiate into all 3 germ layers *in vitro* and *in vivo*. Although gene expression profiles of *in vivo* rPSCs were similar to those of ESCs, they were closer to *in vitro* rPSCs than ESCs. This result is in conflict with reports of *in vivo* rPSCs being closer to ESCs than other iPSCs generated *in vitro* at the transcriptome level^18^. Abad *et al*. found that *in vivo* iPSCs were so potent that they differentiated into the trophoblast lineage^18^. However, we did not observe *in vivo* rPSCs contributing to the trophectoderm of blastocysts after aggregation with 8-cell embryos (Fig. 2G). A recent report showed that only ESCs expressing 2-cell–specific genes had the ability to contribute to both embryonic and extraembryonic tissues^23^. Moreover, ESCs grown in 2i-containing medium had a better potential to differentiate into trophoblast and extraembryonic endoderm than those grown in conventional ESC culture medium^24^. Therefore, the ability of iPSCs to differentiate into the trophoblast lineage might be related more to culture environment than to the *in vivo* milieu. Stable pluripotent stem cells do not reside *in vivo*; instead, they exist transiently during early embryonic development. Stable pluripotent stem cells were found to be established from pre-implantation embryo or post-implantation epiblast cells (5.5–6.5 dpc) were cultured in an *in vitro* system^25^. Therefore, the *in vivo* environment might not always favor pluripotential reprogramming, although pluripotency might be exhibited. Most importantly, trophoblastic differentiation is not a feature of pluripotency in mice. Thus, it is not reasonable to estimate the quality of pluripotent cells because of trophoblastic differentiation potential.

We used homozygous reprogrammable mice for *in vivo* rPSCs because MEFs from heterozygous reprogrammable mice were rarely reprogrammed to *Oct4*-GFP^+^ cells after doxycycline treatment *in vitro*. Only 1 copy of the reprogramming gene set might not be sufficient for the successful reprogramming of MEFs. Reprogramming efficiency is much lower in MEFs containing 1 copy of each transgene (Het/Het) than in MEFs that were homozygous for *OKSM* and *Rosa26-M2rtTA* transgenes^21^. It was recently reported that doxycycline-inducible reprogrammable MEFs by Oct4 and Tet1 could not be reprogrammed in traditional induction medium but were reprogrammed in specific optimal medium condition^26^. Therefore, the doxycycline-inducible reprogrammable system might need at least 2 copies of the gene set or suitable medium conditions for successful reprogramming.

Notably, the *in vivo* rPSCs in this study were derived from reprogrammable mice without doxycycline treatment. Some reprogrammable mice spontaneously formed aggressively growing tumors containing undifferentiated cells^19^. This phenotype might be attributed to the leaky expression of the transgene in an undefined cell type. The underlying mechanism is unclear; however, it is possible that the *in vivo* microenvironment influenced the regulation of doxycycline-inducible transgenes. Recently, we found a clue for the reactivation of integrated transgenes in an *in vitro* system. Integrated reprogramming factor genes, which were inactive in the iPSC state, were spontaneously re-activated when the iPSCs were differentiated into NSCs *in vitro*^27^. The reactivation of transgenes was closely correlated with the change in the levels of DNA methyltransferases during the differentiation of iPSCs. These results indicate that somatic cells could be reprogrammed into pluripotent cells not only *in vitro* but also *in vivo*^19^,^22^.

As shown by *in vivo* iPSC generation through teratoma formation, the process of iPSC derivation shares many characteristics with cancer development. During reprogramming, differentiated somatic cells acquire properties of self-renewal along with unlimited proliferation and exhibit global alterations of the transcriptional program, which are also critical events during carcinogenesis^28^. Partial reprogramming *in vivo* can bring about cancer development^29^,^30^. Ohnishi and colleagues showed that premature termination of reprogramming by transient expression of reprogramming factors led to tumor formation *in vivo*. These tumor cells could be fully reprogrammed into iPSCs by further induction of reprogramming factors. Therefore, tumor formation *in vivo* could be a result of incomplete reprogramming. In the present study, we showed that once completely reprogrammed, *in vivo* rPSCs formed through tumor formation possessed pluripotency and resembled *in vitro* iPSCs, which do not contribute to the trophectoderm.

## Methods

The methods were carried out in accordance with the approved guidelines and all experimental protocols were approved by the Institutional Animal Care and Use Committee of Konkuk University

### Generation of reprogrammable OG2 mice

Homozygous OG2 mice were crossed with homozygous reprogrammable (RTC4) mice, and then heterozygous OG2^+/−^/RTC4^+/−^ mice were crossed with homozygous RTC4 mice. The resulting mice were OG2^+/−^/RTC4^+/+^, OG2^+/−^/RTC4^+/−^, OG2^−/−^/RTC4^+/+^, and OG2^−/−^/RTC4^+/−^. Finally, we selected OG2^+/−^ RTC4^+/+^ mice (rOG2) by genotyping and test crosses with wild-type mice. The primers for genotyping were as follows: GFP sense 5′-GCAAGCTGACCCTGAAGTTCA-3′, GFP antisense 5′-TCACCTTGATGCCGTTCTTCT-3′, OKSM #1 5′-GCACAGCATTGCGGACATG-3′, OKSM #2-1 5′-CCCTCCATGTGTGACCAAGG-3′, OKSM #2-2 5′-CCCTCCATGTGTGACCAAGG-3′, M2rtTA #1 5′-GCGAAGAGTTTGTCCTCAACC-3′, M2rtTA #2 5′-AAAGTCGCTCTGAGTTGTTAT-3′, and M2rtTA #3 5′-GGAGCGGGAGAAATGGATATG-3′. Animals were maintained and used for experimentation under the guidelines of the Institutional Animal Care and Use Committee of Konkuk University.

### Isolation of *in vivo* rPSCs from rOG2 teratomas

Pieces of teratomas were washed and chopped in PBS containing 10 × penicillin/streptomycin/glutamine. Collected tissues were centrifuged at 900 rpm for 3 min. For single-cell dissociation, tissues were pipetted in 0.25% trypsin and incubated at 37 °C for 5 min. This step was repeated thrice. Dissociated tissues were then filtered through a 70 μm mesh cell strainer for single-cell purification.

### Cell culture

GFP positive cells from teratomas were cultured in DMEM supplemented with 15% fetal bovine serum (FBS), 1× penicillin/streptomycin/glutamine, 0.1 mM nonessential amino acids, 1 mM β-mercaptoethanol, and 10^3^ units/ml leukemia inhibitory factors (LIF) on feeder layers.

### RNA isolation and quantitative polymerase chain reaction analysis

Total RNA was isolated using the RNeasy Mini Kit (Qiagen) and treated with DNase to remove genomic DNA contamination. Total RNA (1 μg) was reverse transcribed using the SuperScript III Reverse Transcriptase Kit (Invitrogen) and Oligo(dT) primer (Invitrogen) according to the manufacturer’s instructions. Quantitative polymerase chain reaction (qRT-PCR) reactions were set up in duplicate with the Power SYBR Green Master Mix (Takara) and analyzed with the Roche LightCycler 5480 (Roche). The primers for qRT-PCR used were as follows: *Oct4* (endo) sense, 5′-GATGCTGTGAGCCAAGGCAAG-3′; *Oct4* (endo) antisense, 5′-GGCTCCTGATCAACAGCATCAC-3′; *Nanog* (endo) sense, 5′-CTTTCACCTATTAAGGTGCTTGC-3′; *Nanog* (endo) antisense, 5′-TGGCATCGGTTCATCATGGTAC-3′; *Rex1* sense, 5′-TCCATGGCATAGTTCCACAG-3′; *Rex1* antisense, 5′-TAACTGATTTTCTGCCGTATGC-3′; ACTB sense, 5′-CGCCATGGATGACGATATCG-3′; and ACTB antisense, 5′-CGAAGCCGGCTTTGCACATG-3′.

### Bisulfite genomic sequencing

To differentiate methylated from unmethylated CG dinucleotides, genomic DNA was treated with sodium bisulfite to convert all unmethylated cytosine residues into uracil residues using the EpiTect Bisulfite Kit (Qiagen) according to the manufacturer’s protocol. Briefly, purified genomic DNA (0.5–1 μg) was denatured at 99 °C and then incubated at 60 °C. Modified DNA, i.e., after desulfonation, neutralization, and desalting, was diluted with 20 μl of distilled water. Subsequently, bisulfite PCR (BS-PCR) was carried out using 1–2-μl aliquots of modified DNA for each PCR. The primers used for BS-PCR were as follows: *Oct4* 1st sense, 5′-TTTGTTTTTTTATTTATTTAGGGGG-3′; *Oct4* 1st antisense, 5′-ATCCCCAATACCTCTAAACCTAATC-3′; *Oct4* 2nd sense, 5′-GGGTTGGAGGTTAAGGTTAGAGGG-3′; *Oct4* 2nd antisense, 5′-CCCCCACCTAATAAAAATAAAAAAA-3′; *Nanog* 1st sense, 5′-TTTGTAGGTGGGATTAATTGTGAA-3′; *Nanog* 1st antisense, 5′-AAAAAATTTTAAACAACAACCAAAAA-3′; *Nanog* 2nd sense, 5′-TTTGTAGGTTGGGATTAATTGTGAA-3′; *Nanog* 2nd antisense, 5′-AAAAAAACAAAACACCAACCAAAT-3′.

Briefly, the amplified products were verified by electrophoresis on 1% agarose gel. The desired PCR products were used for subcloning using the TA cloning vector (pGEM-T Easy Vector; Promega). The reconstructed plasmids were purified, and individual clones were sequenced (Solgent Corporation).

### Immunocytochemistry experiments

For immunocytochemistry, cells were fixed with 4% paraformaldehyde for 20 min at room temperature. After cells were washed with PBS, they were treated with PBS containing 10% normal goat serum and 0.03% Triton X-100 for 45 min at room temperature. The primary antibodies used were anti-*Oct4* (*Oct4*; monoclonal, 1:100, Abcam, sc-9081), anti-*nanog* (*nanog*; monoclonal, 1:200, Abcam, ab80892), anti-*Sox2* (*Sox2*; polyclonal, 1:500, Millipore, AB5603), anti-tubulin, beta III (Tuj1; monoclonal, 1:1000, Millipore, MAB1637), anti-SMA (SMA; monoclonal, 1:200, Abcam, ab7817), and Sox17 (Sox17; polyclonal, 1:200, R&D systems, AF1924). For the detection of primary antibodies, fluorescence-labeled secondary antibodies (Alexa fluor 488 or 568; Molecular Probes, Eugene, OR, USA) were used according to the specifications of the manufacturer.

### Flow cytometry

Dissociated cells of teratomas were washed with PBS, and resuspended in ESC medium. GFP-positive cells were sorted directly into ESC medium using FACSAria cell sorter with FACSDiva software.

### Chimera formation

*In vivo* iPSCs were aggregated with denuded post-compacted 8-cell–stage embryos to obtain an aggregate chimera. Eight-cell embryos flushed from 2.5-dpc B6D2F1 female mice were cultured in microdops of embryo culture medium under mineral oil. After cells were trypsinized for 10 s, clumps of iPSCs (4–10 cells) were selected and transferred into microdrops containing zona-free 8-cell embryos. Morula-stage embryos aggregated with iPSCs were cultured overnight at 37 °C and 5% CO_2_. The aggregated blastocysts were transferred into one of the uterine horns of 2.5-dpc pseudopregnant recipients. The primers for genotyping of GFP were as follows: GFP sense 5′-GCAAGCTGACCCTGAAGTTCA-3′, GFP antisense 5′-TCACCTTGATGCCGTTCTTCT-3′, ACTB sense 5′-CGCCATGGATGACGATATCG-3′, and ACTB antisense 5′-CGAAGCCGGCTTTGCACATG-3′.

### Microarray-based analysis

Total RNA was isolated using an RNeasy Mini Kit (Qiagen) and digested with DNase I (RNase-free DNase, Qiagen) according to the manufacturer’s instructions. Total RNA was amplified, biotinylated, and purified using the Ambion Illumina RNA amplification kit (Ambion) according to the manufacturer’s instructions. Biotinylated cRNA samples (750 ng) were hybridized to each MouseRef-8 v2 Expression BeadChip (Illumina), and signals were detected using Amersham fluorolink streptavidin-Cy3 (GE Healthcare Life Sciences) by following the Illumina bead array protocol. Arrays were scanned with an Illumina BeadArray Reader confocal scanning system according to the manufacturer’s instructions.

Raw data were extracted using the software provided by the manufacturer (Illumina GenomeStudio v2011. 1, Gene Expression Module v1.9. 0). Array data were filtered on the basis of p-values of <0.05 in at least 50% of the samples. The selected probe signal value was logarithmically transformed and normalized using the quantile method. Comparative analyses were carried out using the local pooled error test and fold change. False discovery rate was controlled by adjusting the p-values using the Benjamini–Hochberg algorithm. Hierarchical clustering was performed using average linkage and Pearson distance as a measure of similarity.

## Additional Information

**How to cite this article**: Choi, H. W. *et al*. *In vivo* reprogrammed pluripotent stem cells from teratomas share analogous properties with their *in vitro* counterparts. *Sci. Rep*. **5**, 13559; doi: 10.1038/srep13559 (2015).

## Supplementary Material

Supplementary Information

## References

[b1] Nagata S. . Efficient reprogramming of human and mouse primary extra-embryonic cells to pluripotent stem cells. Genes to cells: devoted to molecular & cellular mechanisms 14, 1395–1404, 10.1111/j.1365-2443.2009.01356.x (2009). 19912344

[b2] Kim M. J. . Conversion of genomic imprinting by reprogramming and redifferentiation. Journal of cell science 126, 2516–2524, 10.1242/jcs.122754 (2013). 23525019

[b3] Chin M. H. . Induced pluripotent stem cells and embryonic stem cells are distinguished by gene expression signatures. Cell stem cell 5, 111–123 (2009). 19570518 10.1016/j.stem.2009.06.008 PMC3448781

[b4] Warren L. . Highly efficient reprogramming to pluripotency and directed differentiation of human cells with synthetic modified mRNA. Cell stem cell 7, 618–630, 10.1016/j.stem.2010.08.012 (2010). 20888316 PMC3656821

[b5] Kaji K. . Virus-free induction of pluripotency and subsequent excision of reprogramming factors. Nature 458, 771–775, 10.1038/nature07864 (2009). 19252477 PMC2667910

[b6] Yu J. . Human induced pluripotent stem cells free of vector and transgene sequences. Science 324, 797–801, 10.1126/science.1172482 (2009). 19325077 PMC2758053

[b7] Kim D. . Generation of human induced pluripotent stem cells by direct delivery of reprogramming proteins. Cell stem cell 4, 472–476, 10.1016/j.stem.2009.05.005 (2009). 19481515 PMC2705327

[b8] Zhou H. . Generation of induced pluripotent stem cells using recombinant proteins. Cell stem cell 4, 381–384, 10.1016/j.stem.2009.04.005 (2009). 19398399 PMC10182564

[b9] Hou P. . Pluripotent stem cells induced from mouse somatic cells by small-molecule compounds. Science 341, 651–654, 10.1126/science.1239278 (2013). 23868920

[b10] Miyanari Y. & Torres-Padilla M. E. Control of ground-state pluripotency by allelic regulation of Nanog. Nature 483, 470–473, 10.1038/nature10807 (2012). 22327294

[b11] Ying Q.-L. . The ground state of embryonic stem cell self-renewal. Nature 453, 519–523 (2008). 18497825 10.1038/nature06968 PMC5328678

[b12] Hirano K. . Human and mouse induced pluripotent stem cells are differentially reprogrammed in response to kinase inhibitors. Stem cells and development 21, 1287–1298 (2011). 21882976 10.1089/scd.2011.0283

[b13] Maherali N. & Hochedlinger K. Guidelines and techniques for the generation of induced pluripotent stem cells. Cell stem cell 3, 595–605, 10.1016/j.stem.2008.11.008 (2008). 19041776

[b14] Niu W. . *In vivo* reprogramming of astrocytes to neuroblasts in the adult brain. Nature cell biology 15, 1164–1175 (2013). 24056302 10.1038/ncb2843 PMC3867822

[b15] Torper O. . Generation of induced neurons via direct conversion *in vivo*. Proceedings of the National Academy of Sciences 110, 7038–7043 (2013). 10.1073/pnas.1303829110 PMC3637783 23530235

[b16] Grande A. . Environmental impact on direct neuronal reprogramming *in vivo* in the adult brain. Nature communications 4, 2373, 10.1038/ncomms3373 (2013). PMC3786770 23974433

[b17] Yilmazer A., De Lázaro I., Bussy C. & Kostarelos K. *In Vivo* Cell Reprogramming towards Pluripotency by Virus-Free Overexpression of Defined Factors. PloS one 8, e54754 (2013). 23355895 10.1371/journal.pone.0054754 PMC3552956

[b18] Abad M. . Reprogramming *in vivo* produces teratomas and iPS cells with totipotency features. Nature 502, 340–345, 10.1038/nature12586 (2013). 24025773

[b19] Stadtfeld M., Maherali N., Borkent M. & Hochedlinger K. A reprogrammable mouse strain from gene-targeted embryonic stem cells. Nature methods 7, 53–55 (2009). 20010832 10.1038/nmeth.1409 PMC3987893

[b20] Li Y. . BMP4-directed trophoblast differentiation of human embryonic stem cells is mediated through a ΔNp63+ cytotrophoblast stem cell state. Development 140, 3965–3976 (2013). 24004950 10.1242/dev.092155 PMC3775414

[b21] Polo J. M. . A molecular roadmap of reprogramming somatic cells into iPS cells. Cell 151, 1617–1632, 10.1016/j.cell.2012.11.039 (2012). 23260147 PMC3608203

[b22] Markoulaki S. . Transgenic mice with defined combinations of drug-inducible reprogramming factors. Nature biotechnology 27, 169–171 (2009). 10.1038/nbt.1520 PMC2654270 19151700

[b23] Macfarlan T. S. . Embryonic stem cell potency fluctuates with endogenous retrovirus activity. Nature 487, 57–63 (2012). 22722858 10.1038/nature11244 PMC3395470

[b24] Morgani S. M. . Totipotent embryonic stem cells arise in ground-state culture conditions. Cell reports 3, 1945–1957 (2013). 23746443 10.1016/j.celrep.2013.04.034 PMC3701323

[b25] Boroviak T., Loos R., Bertone P., Smith A. & Nichols J. The ability of inner-cell-mass cells to self-renew as embryonic stem cells is acquired following epiblast specification. Nature cell biology 16, 516–528, 10.1038/ncb2965 (2014). 24859004 PMC4878656

[b26] Chen J. . The combination of tet1 with oct4 generates high-quality mouse-induced pluripotent stem cells. Stem cells 33, 686–698, 10.1002/stem.1879 (2015). 25331067

[b27] Choi H. W. . Neural stem cells differentiated from iPS cells spontaneously regain pluripotency. Stem cells 32, 2596–2604, 10.1002/stem.1757 (2014). 24898298

[b28] Ben-Porath I. . An embryonic stem cell–like gene expression signature in poorly differentiated aggressive human tumors. Nature genetics 40, 499–507 (2008). 18443585 10.1038/ng.127 PMC2912221

[b29] Hobbs R. M. & Polo J. M. Reprogramming Can Be a Transforming Experience. Cell stem cell 14, 269–271 (2014). 24607400 10.1016/j.stem.2014.02.003

[b30] Ohnishi K. . Premature termination of reprogramming *in vivo* leads to cancer development through altered epigenetic regulation. Cell 156, 663–677 (2014). 24529372 10.1016/j.cell.2014.01.005

