# Supplementary material for: In vivo reprogrammed pluripotent stem cells from teratomas share analogous properties with their in vitro counterparts
Source: Sci Rep. 2015 Aug 28;5:13559. doi: 10.1038/srep13559 (PMC4551988; doi:10.1038/srep13559)
Supplement: Supplementary Information [file srep13559-s1.pdf]

Supplementary information

In vivo reprogrammed pluripotent stem cells from teratomas share analogous properties with their *in vitro* counterparts

Hyun Woo Choi, Jong Soo Kim, Yean Ju Hong, Hyuk Song, Han Geuk Seo, and Jeong Tae Do

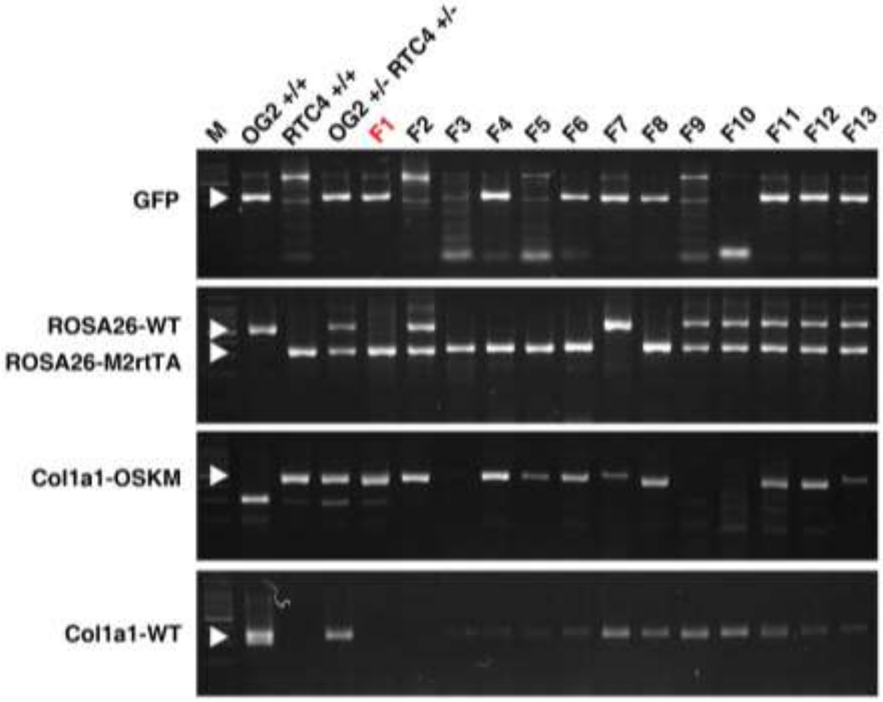

Figure S1. Genotyping of *Oct4*-GFP, reprogramming gene (Oct4, Klf4, Sox2, and c-Myc), and reverse tetracycline-controlled transactivator (rtTA) by PCR. The founder 1 (F1) mouse is homozygous for the transcriptional activator (ROSA26-M2rtTA) and doxycycline-inducible polycistronic cassette encoding the 4 reprogramming factors OSKM within the Col1a1 locus, and heterozygous for Oct4-GFP transgene (ΔPE). This F1 is named as rOG2 (for reprogrammable OG2) mouse.

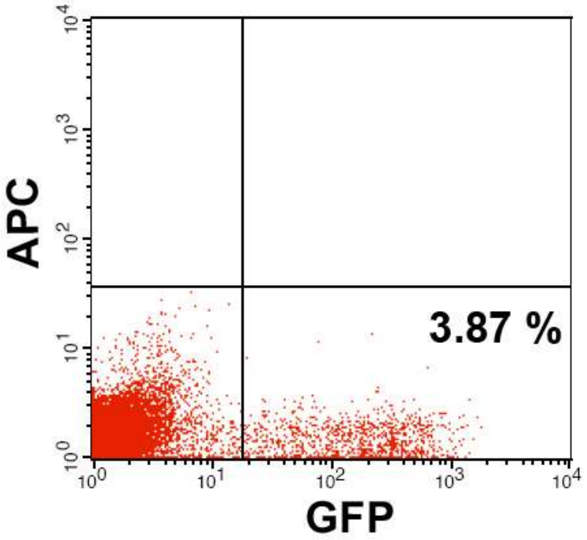

Figure S2. FACS analysis of the teratomas from rOG2 mouse. Teratomas formed in rOG2 mouse contain about 3.87% ± 0.02 (mean ± SD) of the *Oct4*-GFP positive cells.

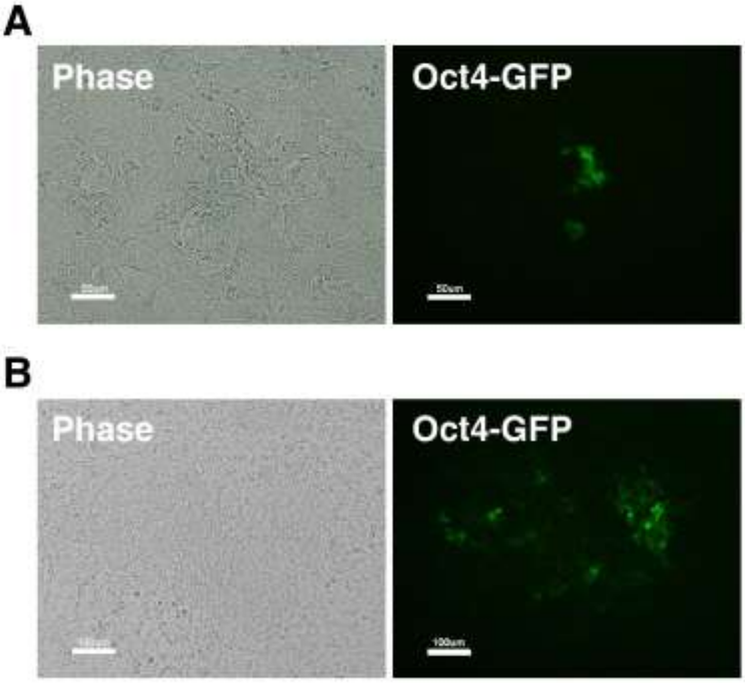

Figure S3. Reprogramming of rOG2 somatic cells. NSCs (A) and MEFs (B) could be reprogrammed *in vitro* into pluripotent *Oct4*-GFP$^+$ cells by treatment of doxycycline. *Oct4*-GFP$^+$ colonies were observed at day 13 and 15, respectively. Scale bar = 50 and 100 μm

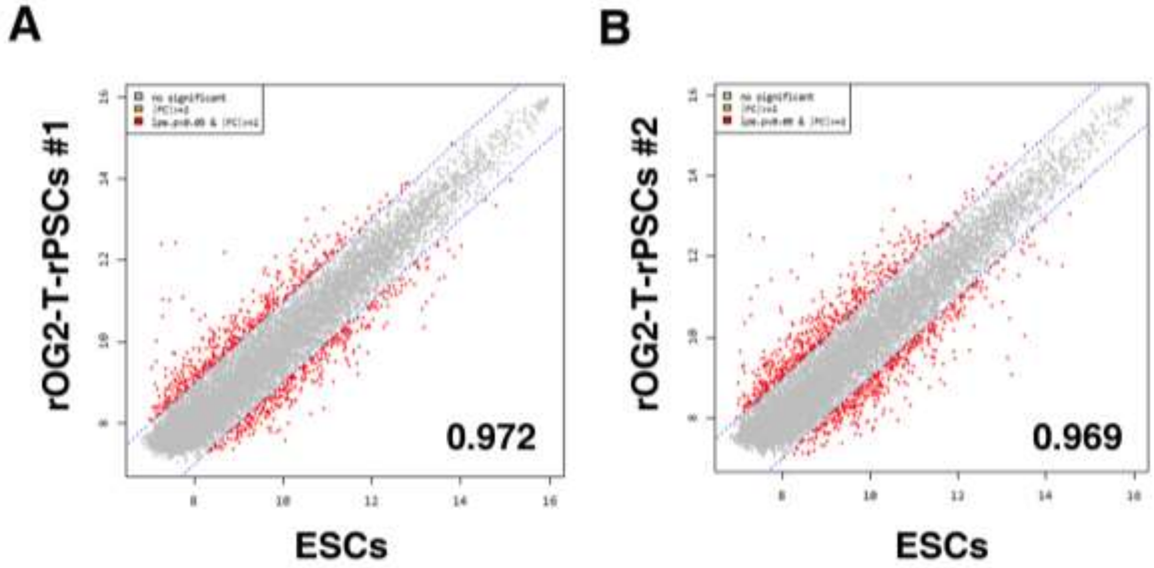

Figure S4. Similarity of the gene expression patterns between ESCs and rOG2-T-iPSCs #1 and #2.

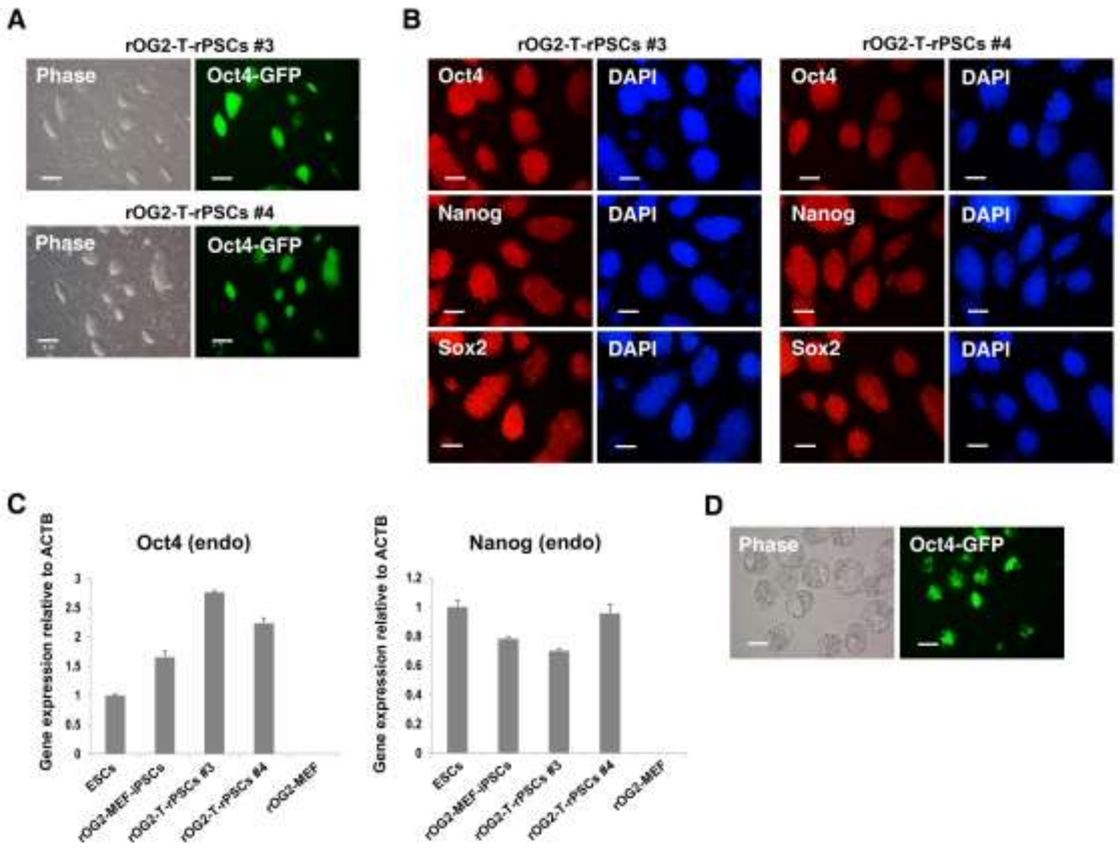

Figure S5. Characterization of rOG2-T-rPSCs #3 and #4. (A) Phase and fluorescence microscope images of rOG2-T-rPSCs #3 and #4. (B) Immuncytochemistry experiment for Oct4, Nanog, and Sox2 in rOG2-T-rPSCs #3 and #4. (C) Real time RT-PCR showed that rOG2-T-rPSCs #3 and #4 expressed Oct4 and Sox2 like ESCs. (D) rOG2-T-rPSCs #3 could incorporate into ICM of blastocyst, but not trophectoderm.

Supplemetary Table 1. Gene ontology analysis of the upregulated gene in rOG2-T-iPSCs

**Annotation Cluster 1**	**Enrichment Score: 2.63**			
**GO term**	**Count**	**Genes**	**P_Value**	**Benjamini**
apoptosis	20	IRAK2, BCL10, E2F2, PDCD11, PDCD10, TNFRSF12A, PGLYRP1, PIM2, DIDO1, BCL2L11, PPM1F, BNIPL, BOK, ID1, KRT8, ACIN1, GADD45B, DEDD2, PHLDA1, PEG3	2.00E-03	4.50E-01
cell death	21	IRAK2, BCL10, E2F2, KLK8, PDCD11, PDCD10, TNFRSF12A, PGLYRP1, PIM2, DIDO1, BCL2L11, PPM1F, BNIPL, BOK, ID1, KRT8, ACIN1, GADD45B, DEDD2, PHLDA1, PEG3	2.30E-03	4.40E-01
programmed cell death	20	IRAK2, BCL10, E2F2, PDCD11, PDCD10, TNFRSF12A, PGLYRP1, PIM2, DIDO1, BCL2L11, PPM1F, BNIPL, BOK, ID1, KRT8, ACIN1, GADD45B, DEDD2, PHLDA1, PEG3	2.40E-03	4.10E-01
death	21	IRAK2, BCL10, E2F2, KLK8, PDCD11, PDCD10, TNFRSF12A, PGLYRP1, PIM2, DIDO1, BCL2L11, PPM1F, BNIPL, BOK, ID1, KRT8, ACIN1, GADD45B, DEDD2, PHLDA1, PEG3	3.00E-03	3.40E-01

**Annotation Cluster 2**	**Enrichment Score: 2.17**			
**GO term**	**Count**	**Genes**	**P_Value**	**Benjamini**
cell cycle	30	E2F2, S100A6, SEPT1, CCNT1, PTTG1, CHEK2, SESN1, SPC24, CCNE1, RSPH1, OVOL1, TXNIP, NASP, AK1, 1190002H23RIK, LOC100045040, MYH9, KLHDC3, NCAPD2, CCND1, CDKN1A, RCC2, RASSF1, NUDC-PS1, CALM3, AKAP8, RUVBL1, SMC1A, SEPT6, TXNL4A	8.70E-06	1.30E-02
cell division	15	SEPT1, CCNT1, PTTG1, MYH9, NCAPD2, SPC24, CCNE1, CDC42, CCND1, RCC2, NUDC-PS1, RUVBL1, SMC1A, SEPT6, TXNL4A	1.30E-03	6.20E-01

cell cycle process	18	AK1, PTTG1, MYH9, SESN1, KLHDC3, NCAPD2, SPC24, CCND1, CDKN1A, RCC2, RSPH1, RASSF1, OVOL1, NUDC-PS1, RUVBL1, AKAP8, SMC1A, TXNL4A	1.80E-03	5.10E-01
M phase	13	PTTG1, MYH9, NCAPD2, KLHDC3, SPC24, RCC2, RSPH1, OVOL1, NUDC-PS1, RUVBL1,<br>AKAP8, SMC1A, TXNL4A	9.80E-03	5.10E-01

**Annotation Cluster 3**	**Enrichment Score: 1.76**			
**GO term**	**Count**	**Genes**	**P_Value**	**Benjamini**
angiogenesis	9	BMP4, RTN4, PDGFA, TNFRSF12A, EDN1, PLCD1, MYH9, SRF, THY1	4.50E-03	3.90E-01

**Annotation Cluster 4**	**Enrichment Score: 1.57**			
**GO term**	**Count**	**Genes**	**P_Value**	**Benjamini**
hexose catabolic process	6	ALDOA, LDHC, TPI1, PGD, PGAM1, MDH2	3.30E-03	3.50E-01
glucose catabolic process	6	ALDOA, LDHC, TPI1, PGD, PGAM1, MDH2	3.30E-03	3.50E-01
monosaccharide catabolic process	6	ALDOA, LDHC, TPI1, PGD, PGAM1, MDH2	3.90E-03	3.70E-01
carbohydrate catabolic process	7	ALDOA, LDHC, TPI1, PGD, PGAM1, PGLYRP1, MDH2	5.00E-03	4.00E-01
cellular carbohydrate catabolic process	6	ALDOA, LDHC, TPI1, PGD, PGAM1, MDH2	6.20E-03	4.30E-01
alcohol catabolic process	6	ALDOA, LDHC, TPI1, PGD, PGAM1, MDH2	8.70E-03	5.10E-01

**Annotation Cluster 5**	**Enrichment Score: 1.56**			
**GO term**	**Count**	**Genes**	**P_Value**	**Benjamini**
M phase	13	PTTG1, MYH9, NCAPD2, KLHDC3, SPC24, RCC2, RSPH1, OVOL1, NUDC-PS1, RUVBL1,<br>AKAP8, SMC1A, TXNL4A	9.80E-03	5.10E-01

Supplemetary Table 2. Gene ontology analysis of the downregulated gene in rOG2-T-iPSCs

Annotation Cluster 1	Enrichment Score: 1.86			
**GO term**	**Count**	**Genes**	**P_Value**	**Benjamini**
cell redox homeostasis	7	GLRX3, TXNDC12, AIFM1, DLD, PDIA6, TXN1, DDIT3	4.30E-04	4.50E-01

Supplemetary Table 3. KEGG pathway analysis of upregualted genes in rOG2-T-iPSCs

KEGG_PATHWAY	Count	Genes	P-Value	Benjamini
p53 signaling pathway	8	CCNE1, CDKN1A, CCND1, RRM2, SFN, CHEK2, GADD45B, SESN1	6.00E-04	6.80E-02
Cell cycle	10	CCNE1, E2F2, CDKN1A, CCND1, YWHAG, PTTG1, SFN, CHEK2, GADD45B, SMC1[illegible]	1.50E-03	8.50E-02

Supplemetary Table 4. KEGG pathway analysis of downregualted genes in rOG2-T-iPSCs

KEGG_PATHWAY	Count	Genes	P-Value	Benjamini
Propanoate metabolism	5	ALDH6A1, ACSS1, ACSS2, PCCB, ALDH3A2	8.00E-04	8.20E-02
Pyruvate metabolism	5	ACSS1, AKR1B3, DLD, ACSS2, ALDH3A2	2.60E-03	1.30E-01
Glycolysis / Gluconeogenesis	6	ACSS1, DLD, ENO2, ACSS2, ALDH3A2, ENO1	2.70E-03	9.20E-02
Valine, leucine and isoleucine degradation	5	ALDH6A1, DLD, PCCB, ALDH3A2, HADHB	4.00E-03	1.00E-01
